# The feasibility of half-dose contrast-enhanced scanning of brain tumours at 5.0 T: a preliminary study

**DOI:** 10.1186/s12880-024-01270-z

**Published:** 2024-04-13

**Authors:** Zhiyong Jiang, Wenbo Sun, Dan Xu, Hao Mei, Jianmin Yuan, Xiaopeng Song, Chao Ma, Haibo Xu

**Affiliations:** 1 Department of Radiology, Zhongnan Hospital of Wuhan University, Wuhan, China; 2 Medical Imaging Department, Shenzhen Ban’an Traditional Chinese Medicine Hospital Group, Shenzhen, China; 3 Department of Nuclear Medicine, Zhongnan Hospital of Wuhan University, Wuhan, China; 4 United Imaging Healthcare, Shanghai, China; 5 Wuhan Zhongke Industrial Research Institute, Wuhan, Hubei China; 6 Department of Neurosurgery, Zhongnan Hospital, Wuhan, China

**Keywords:** 5.0 T, MRI, Half-dose contrast agent, Ultrahigh field, Brain tumours

## Abstract

**Purpose:**

This study investigated and compared the effects of Gd enhancement on brain tumours with a half-dose of contrast medium at 5.0 T and with a full dose at 3.0 T.

**Methods:**

Twelve subjects diagnosed with brain tumours were included in this study and underwent MRI after contrast agent injection at 3.0 T (full dose) or 5.0 T (half dose) with a 3D T1-weighted gradient echo sequence. The postcontrast images were compared by two independent neuroradiologists in terms of the signal-to-noise ratio (SNR), contrast-to-noise ratio (CNR) and subjective image quality score on a ten-point Likert scale. Quantitative indices and subjective quality ratings were compared with paired Student's t tests, and interreader agreement was assessed with the intraclass correlation coefficient (ICC).

**Results:**

A total of 16 enhanced tumour lesions were detected. The SNR was significantly greater at 5.0 T than at 3.0 T in grey matter, white matter and enhanced lesions (*p* < 0.001). The CNR was also significantly greater at 5.0 T than at 3.0 T for grey matter/tumour lesions, white matter/tumour lesions, and grey matter/white matter (*p* < 0.001). Subjective evaluation revealed that the internal structure and outline of the tumour lesions were more clearly displayed with a half-dose at 5.0 T (Likert scale 8.1 ± 0.3 at 3.0 T, 8.9 ± 0.3 at 5.0 T, *p* < 0.001), and the effects of enhancement in the lesions were comparable to those with a full dose at 3.0 T (7.8 ± 0.3 at 3.0 T, 8.7 ± 0.4 at 5.0 T, *p* < 0.001). All subjective scores were good to excellent at both 5.0 T and 3.0 T.

**Conclusion:**

Both quantitative and subjective evaluation parameters suggested that half-dose enhanced scanning via 5.0 T MRI might be feasible for meeting clinical diagnostic requirements, as the image quality remains optimal. Enhanced scanning at 5.0 T with a half-dose of contrast agents might benefit patients with conditions that require less intravenous contrast agent, such as renal dysfunction.

## Introduction

Magnetic resonance imaging (MRI) plays a vital role in the diagnosis and characterization of brain tumours due to its high spatial resolution and optimal soft-tissue contrast [1]. However, the key challenge in detecting tumours at an early stage by conventional MRI is its low sensitivity [2]. Therefore, various contrast agents have been developed to improve the sensitivity of MRI [3]. Gadolinium (Gd)-based contrast agents, including Gd-DTPA (Magnevist®), Gd-DO3A-butrol (Gadovist®) and Gd-EOB-DTPA (Primovist®), are the most commonly used contrast agents in clinical practice. Following intravenous administration, Gd-based contrast agents may increase MRI sensitivity for the detection of brain tumours [2, 3]. However, Gd3 + chelates still have low toxicity. The risk of nephrogenic systemic fibrosis (NSF) for patients with impaired renal function and long-term adverse effects due to Gd brain deposition has raised concerns [4–6], especially for patients with brain diseases that require longitudinal monitoring after treatment, such as low-grade gliomas or brain metastases. In diagnosing and treating most brain tumours, multiple gadolinium contrast agent-enhanced scans are required to determine the tumour grade, progression, and prognosis during follow-up. Furthermore, the effects of gadolinium deposits in the brain are still unknown; therefore, doses should be kept as low as possible to prevent gadolinium buildup [7]. Thus, designing new Gd3 + chelates and using lower doses of contrast agents have always been popular research topics [2, 8, 9].

The development of high- and ultrahigh-field MRI scanners (≥ 3.0 Tesla (T)) offers the possibility for using fewer contrast agents in the clinic [9–11]. Many researchers have conducted comparative studies on injection doses at 1.5 T versus 3.0 T and 3.0 T versus 7.0 T and have shown the feasibility of injecting a reduced amount of contrast agents during higher-field MRI for brain tumours [11, 12]. However, whole-body imaging at 7.0 T has not yet been approved by the Food and Drug Administration (FDA) and is not available in the clinic due to safety issues such as increased specific absorption rate (SAR) distributions in the body and technical issues such as B1 field inhomogeneity [13, 14]. Recently, a 5.0 T clinical MRI scanner was developed that can be used to scan the whole body with good image homogeneity and contrast uniformity while avoiding issues such as a high SAR [15]. Many basic MRI applications could benefit from the increased signal intensity (SI), contrast and spatial resolution of 5.0 T systems. However, the feasibility and image quality of lower-dose contrast-enhanced scanning with 5.0 T systems have not been investigated or compared with those of standard full-dose contrast-enhanced scanning with 3.0 T systems.

This study aimed to compare the enhancing effects of half-dose enhanced scanning at 5.0 T and full-dose enhanced scanning at 3.0 T in brain tumours. The enhancement effects were assessed using quantitative indices, including the signal-to-noise ratio (SNR) and contrast-to-noise ratio (CNR), differences in SI before and after enhanced scans, as well as subjective image quality scores. Then, the feasibility of using half-dose contrast agents on 5.0 T MRI for brain tumour diagnosis was evaluated.

## Materials and methods

### Patient characteristics

**Fig. 1:**
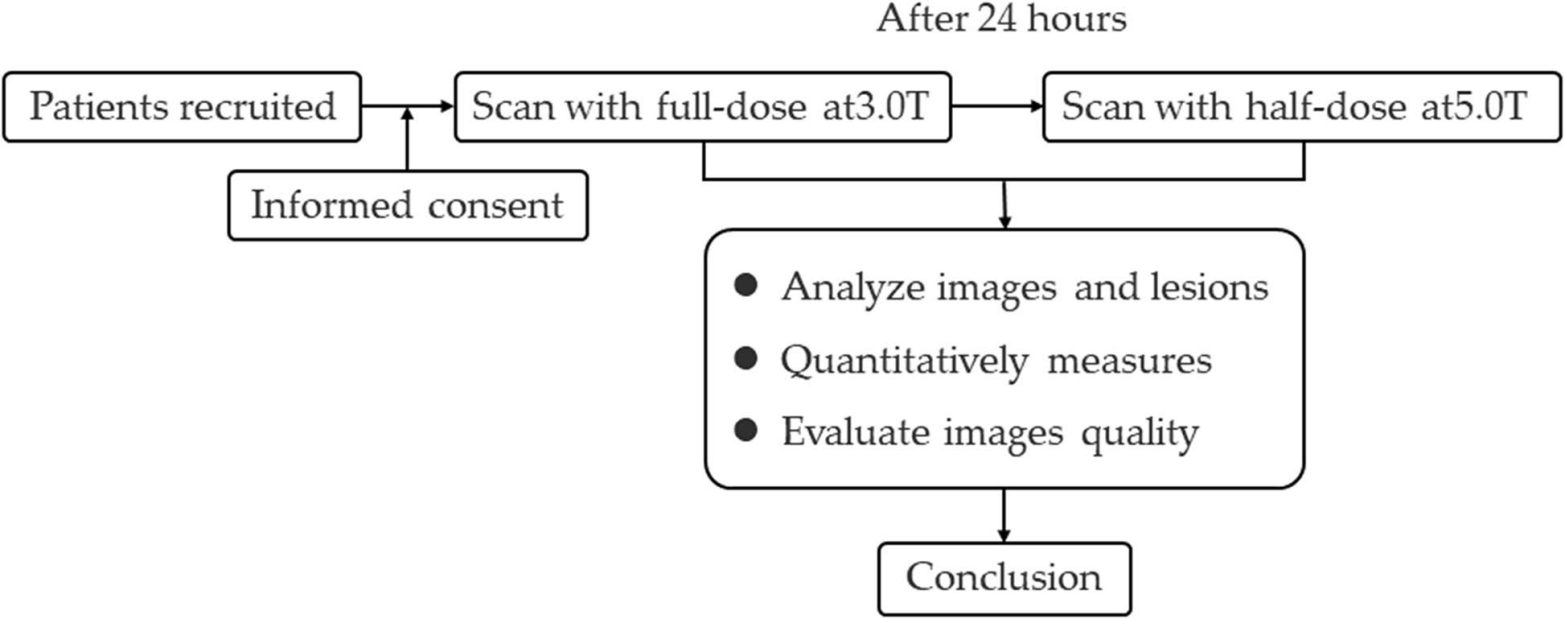
Study design flow diagram

This prospective study was approved by the ethics committee of our hospital (approval no. 2021110). Between 11/2021 and 01/2023, patients suspected of having brain tumours (gliomas, meningiomas or brain metastases) were enrolled, and all participants signed an informed consent form. The inclusion criteria were as follows: 1. 18–80 years old; 2. suspected brain tumours; 3. no safety-related contraindications to undergoing magnetic resonance imaging (MRI) or receiving intravenous contrast agent; and 4. glomerular filtration rate (GFR) > 60 mL/min. The exclusion criterion was poor image quality so that subjective evaluation and image analysis could not be carried out. The participants included in this study underwent both 3.0 T and 5.0 T MRI scans (time interval between the two scans >  = 24 h). The study flowchart is shown in Fig. 1. Finally, a total of 12 patients with clinically diagnosed brain tumours were recruited.

### Image acquisition

All MR scans were performed on a clinical 3.0 T scanner (uMR 790, United Imaging Healthcare) and a clinical 5.0 T scanner (uMR Jupiter, United Imaging Healthcare), with a 48-channel head coil on 5.0 T and a 24-channel head coil on 3.0 T, respectively. A 3D gradient echo (3D-GRE) sequence was acquired in the sagittal plane and reconstructed in the axial and coronal planes. The axial images were used for analysis. A clinically acceptable acquisition time (approximately three and a half min) was used at both 3.0 T and 5.0 T. Under the constraint of this short scanning duration, the sequence parameters were adjusted and optimized to meet the clinical resolution limits at both 3.0 T (0.9 × 0.9 × 0.9 mm^3^) and 5.0 T (0.7 × 0.7 × 0.7 mm^3^) (Table 1). Acceleration techniques such as partial-Fourier k-space filling; compression sensing; and parallel RF transmission (pTx for 5.0 T) and parallel acquisition, which are similar to GRAPPA and SENSE [16], were used for both 3.0 T and 5.0 T imaging.

**Table 1:** Sequence parameters for the 3D gradient echo sequence at 3.0 T and 5.0 T

**Sequence parameters**	**3.0 T**	**5.0 T**
TR/TE/TI	7.6/3.3/810 ms	8.1/2.6/940 ms
FOV	220 × 244 mm^2^	220 × 248 mm^2^
Matrix size	245 × 272	312 × 352
In-plane resolution	0.90 × 0.90 mm^2^	0.70 × 0.70 mm^2^
Slice thickness	0.9 mm	0.7 mm
Flip angle	9°	9°
Number of slices	186	208
Receiver bandwidth	230 Hz/pixel	180 Hz/pixel
Acceleration factor	3.0	3.5
Averages	1	1
Postinjection time	60 s	60 s
Total acquisition duration	204 s	224 s

*TR* repetition time, *TE* echo time, *TI* inversion time, *FOV* field of view

The sequence parameters at 5.0 T were as follows: Repetition time(TR)/Echo time(TE)/Inversion time(TI) = 8.1/2.6/940 ms; FOV = 220 × 248 mm^2^; matrix size = 312 × 352; in-plane resolution = 0.70 × 0.70 mm^2^; slice thickness = 0.7 mm; flip angle = 9°; number of slices = 208; receiver bandwidth = 180 Hz/pixel; acceleration factor = 3.5; averages = 1; and total acquisition duration = 224 s.

The sequence parameters for 3.0 T were as follows: TR/TE/TI = 7.6/3.3/810 ms; FOV = 220 × 244 mm^2^; matrix size = 245 × 272; in-plane resolution = 0.90 × 0.90 mm^2^; slice thickness = 0.9 mm; flip angle = 9°; number of slices = 186; receiver bandwidth = 230 Hz/pixel; acceleration factor = 3.0; averages = 1; and total acquisition duration = 204 s.

The contrast medium administration protocol was as follows: all subjects first underwent intravenous gadolinium contrast-enhanced scans under 3.0 T MRI, with an injection dose of 0.10 mmol/kg (full dose). Gd-DO3A-butrol (Gadovist, Bayer Schering Pharma AG) contrast medium was used. Using a high pressure injector (Insight M12, Jusha Display Technology Co., Ltd., Nanjing) that was 5.0T compliant, the contrast medium was automatically injected at 5.0T. Gadolinium-based MRI contrast agents serve as catalysts for water proton relaxation, and their efficacy is quantified by a rate constant referred to as relaxivity [17]. Gadovist is eliminated from plasma with a mean terminal half-life of 1.81 h (range 1.33–2.13 h) [18]. After 24 h, another contrast-enhanced scan was performed at 5.0 T, with an injection dose of 0.05 mmol/kg (half dose).

### Image analysis and evaluation

The regions of interest (ROIs) were delineated for each subject at locations with normal grey matter and white matter tissues and enhanced tumour lesions with the interactive software tool ITK-SNAP. The in plane size of ROIs placed on the turmous lesions were around 25 to 100 mm^2^. The ROIs of grey matter and white matter were drawn on the slice of the corpus callosum. When drawing the ROIs of the tumours, to ensure the comparability of the measurement data, all the ROIs were placed in the same position (grey matter, white matter and lesion) for measurement on 3.0 T and 5.0 T images by one neuroradiologist (Dr. Jiang, who has 10 years of experience). In instances where there was a discrepancy in image quality between the 3.0 T and 5.0 T images, we employed 3D reconstruction postprocessing techniques to align the results and ensure that the delineated regions of interest (ROIs) were consistently positioned. The quantitative indices, including the SNR and CNR, were calculated as follows.

1 $${{\text{SNR}}}_{tissue}=\frac{{SI}_{tissue}}{{SD}_{background}}$$

2 $${{\text{CNR}}}_{Lesion}=\frac{\left|{SI}_{Lesion}-{SI}_{g/w}\right|}{{SD}_{background}}$$

where ${SI}_{tissue}$ is the SI of tissues (grey matter, white matter and tumour lesions), ${SD}_{background}$ is the standard deviation of SI at the four corners of the background, ${SI}_{Lesion}$ is the SI of tumour tissues, and SIg/w is the SI of grey matter or white matter on the contralateral side. All the calculations were completed in MATLAB (2018a, MathWorks), while the open-source toolbox “*MRIqual*” was used to calculate the SNR (https://github.com/elayden/MRIqual).

We measured the SI of the grey matter, white matter and lesions at 3.0 T and 5.0 T with the same ROI in the T1 image before and after contrast agent injection, subtracted the SI before enhancement from the SI after enhancement, and analysed the changes in SI after enhancement. Concurrently, we assessed the dimensions of the lesions.

The subjective evaluation was performed by two independent neuroradiologists (Dr. Yu, who has 6 years of experience, and Dr. Jiang, who has 10 years of experience). During the evaluation, the MRI scanner information and patient name were anonymized. A ten-point Likert scale was used, and the following parameters were evaluated: lesion-related parameters (contrast agent enhancement, internal structure, and delineation), image quality (grey‒white differentiation and homogeneity), artefacts (motion artefacts, vessel pulsation artefacts, and susceptibility artefacts) and overall sequence quality [11]. All subjective evaluations pertain to the quality of images.

To compare differences in quantitative indices and subjective image quality scores between 3.0 T and 5.0 T scans, paired Student's t tests were conducted. Interreader agreement of the subjective evaluation parameters was assessed by using the intraclass correlation coefficient (ICC) with a two-way random effects model. All the statistical analyses were performed using SPSS 24.0 for Windows. *P* value less than 0.05 was considered to indicate statistical significance.

## Results

### Patient characteristics

As shown in Table 2, the mean age of the participants was 53.58 ± 11.65 years (range: 35–74), with 8 males and 4 females. The mean height was 1.67 ± 0.62 m, the mean weight was 64.23 ± 4.91 kg, and the mean body mass index (BMI) was 22.91 ± 1.17 kg/m^2^. The diagnosis of brain tumours was based on the World Health Organization (WHO) 2016 guidelines [19]. The clinical diagnosis results were as follows: four cases of newly diagnosed meningioma; two cases of newly diagnosed astrocytoma; two cases of suspected brain metastasis (metastatic squamous cell carcinoma of lung origin); one case of anaplastic oligodendroglioma (WHO grade III) recurrence after 7 years of surgery; one case of glioblastoma (WHO grade IV) recurrence 3 years after surgery; one case of left temporal occipital lobe brain tumour (Patient 8, suspected glioblastoma,  with nonsurgical treatment); and one case of left occipital brain tumour (Patient 11, suspected glioblastoma, with nonsurgical treatment). A total of 16 lesions were identified across 12 patients.

**Table 2:** Clinical-pathological characteristics of the patient cohort

**Patient number**	**Age**	**Sex**	**Clinical or histological diagnosis**	**WHO**	**Surgical status**
**Case 1**	35	M	Atypical meningioma	II	First
**Case 2**	49	M	Anaplastic oligodendroglioma	III	Recurrence
**Case 3**	64	M	Brain metastases (lung origin)	/	Non
**Case 4**	43	F	Meningioma	I	First
**Case 5**	64	M	Obese astrocytoma	II	First
**Case 6**	55	M	Meningioma	I	First
**Case 7**	43	F	Glioblastoma	IV	Recurrence
**Case 8**	74	M	Glioblastoma	/	Non
**Case 9**	51	M	Brain metastases (lung origin)	/	Non
**Case 10**	49	F	Astrocytoma	IV	Recurrence
**Case 11**	68	M	Glioblastoma	/	Non
**Case 12**	48	F	Meningioma	I	First

### SNR and CNR

**Fig. 2:**
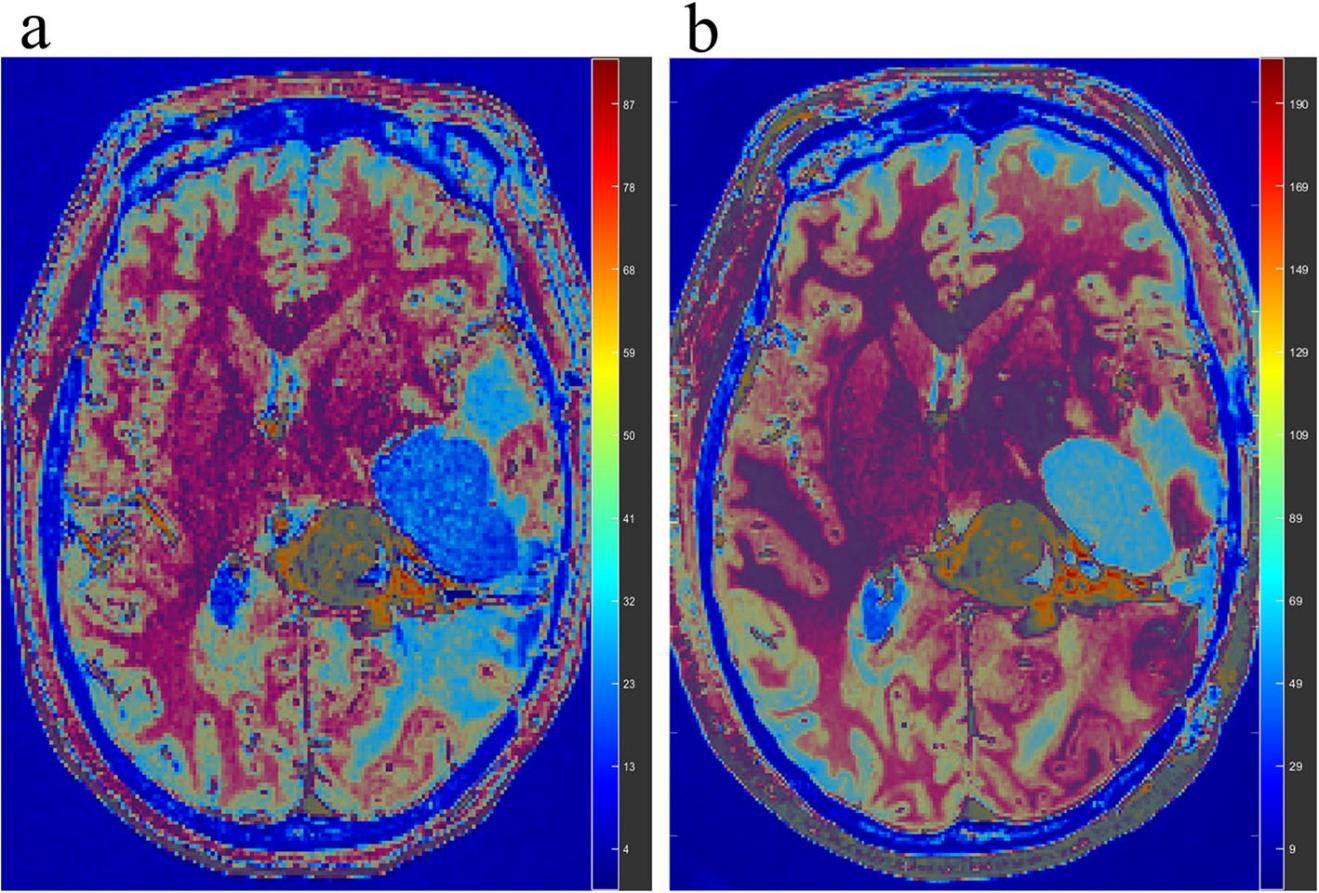
Representative image of a glioma patient (49 years old, female, Patient 10) at 3.0 T (**a**) and 5.0 T (**b**). The 5.0 T images showed a greater SNR than the 3.0 T images, especially for the tumour lesions

A total of 16 enhanced tumour lesions were detected and compared between 3.0 T and 5.0 T images. As shown in Table 3, for tumour lesions, the SNR was 30 ± 8 at 3.0 T and 48 ± 15 at 5.0 T (*P* < 0.001); for white matter, the SNR was 18 ± 3 at 3.0 T and 18 ± 3 at 5.0 T (*P* < 0.001); for grey matter, the SNR was 11 ± 2 at 3.0 T and 22 ± 5 at 5.0 T (*P* < 0.001). The CNR for lesion/white matter was 36 ± 18 at 3.0 T and 69 ± 43 at 5.0 T (*P* < 0.001); the CNR for lesion/grey matter was 40 ± 17 at 3.0 T and 103 ± 49 at 5.0 T (*P* < 0.001); and the CNR for grey matter/white matter was 15 ± 2 at 3.0 T and 33 ± 4 at 5.0 T (*P* < 0.001). The SNR of the tumour lesions was significantly greater with a half-dose at 5.0 T than with a full dose at 3.0 T (Fig. 2).

**Table 3:** SNR, CNR and SI at 3.0 T with a full-dose and at 5.0 T with a half-dose

	**3.0 T**	**5.0 T**	*P* value
**SNR**			
Lesion	30 ± 8	48 ± 15	< 0.001
White matter	18 ± 3	30 ± 5	< 0.001
Grey matter	11 ± 2	22 ± 5	< 0.001
**CNR**			
Lesion/White matter	36 ± 18	69 ± 43	< 0.001
Lesion/Grey matter	40 ± 17	103 ± 49	< 0.001
Gray matter/White matter	15 ± 2	33 ± 4	< 0.001
**SI difference**			
Lesion	430 ± 180	675 ± 333	< 0.001
White matter	44 ± 11	51 ± 11	0.068
Grey matter	43 ± 12	48 ± 13	0.115

*SNR* signal-to-noise ratio, *CNR* contrast-to-noise ratio, *SI difference* postcontrast SI – precontrast SI

### SI difference between postcontrast and precontrast images

**Fig. 3:**
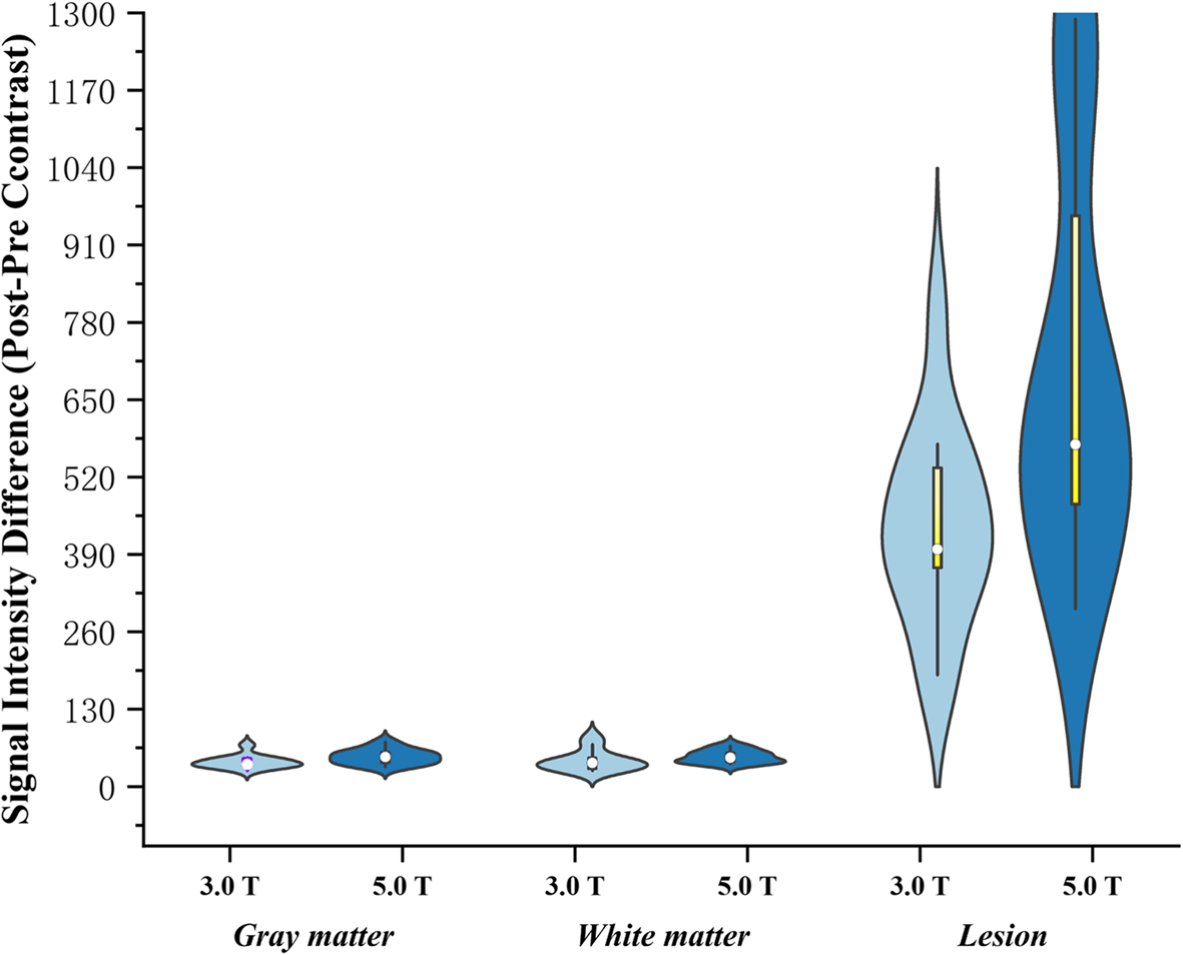
Distribution of SI differences (postcontrast SI—precontrast SI). In grey matter and white matter, there was no significant difference in SI, and in tumour lesions, the difference in SI in the 5.0 T group was significantly greater than that in the 3.0 T group. The yellow line represents the 25%-75% confidence interval. The yellow line depicts a range within the 1.5 interquartile range. The white dot represents the median

The difference distribution diagram of the SI before and after enhancement is shown in Fig. 3. When comparing the SI differences before and after Gd enhancement scans between 3.0 T and 5.0 T, there was no significant difference in white matter or grey matter (white matter, *P* = 0.068 ;gray matter, *P* = 0.115), while the difference was obvious in tumour lesions (*P* < 0.001) (Table 3).

### Subjective evaluation

**Fig. 4:**
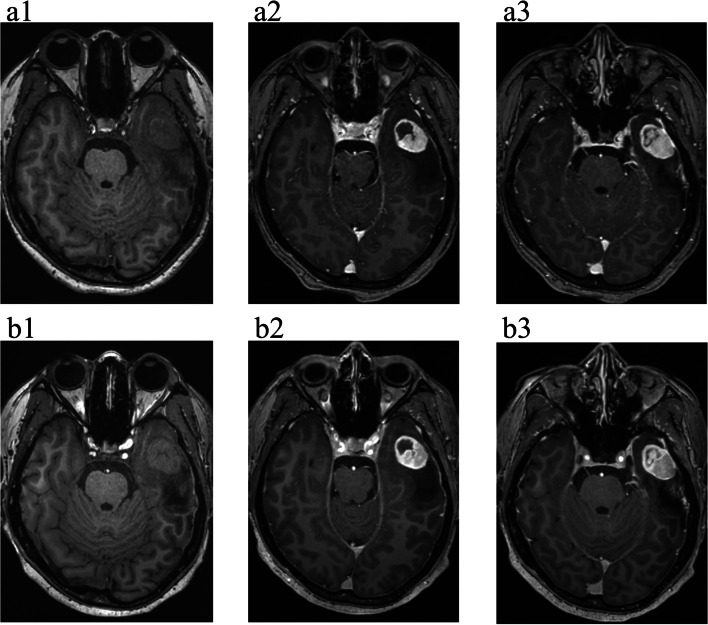
Atypical meningioma, WHO Grade II, 35-year-old male. (**a1**) T1w MRI without contrast enhancement at 3.0 T and (**b1**) T1w MRI without contrast enhancement at 5.0 T. (**a2**) and (**a3**) Two different slices of full-dose enhanced T1w MRI at 3.0 T. (**b2**) and (**b3**) Two different slices of half-dose enhanced T1w MRI at 5.0 T. A 5.0 T MRI displayed a clearer boundary and internal structure of the lesion and showed a stronger inhibitory effect than did 3.0 T MRI; at the same time, in the half-dose 5.0 T images, the degree and range of edge enhancement in the lesion were stronger than those in the full-dose 3.0 T images

**Fig. 5:**
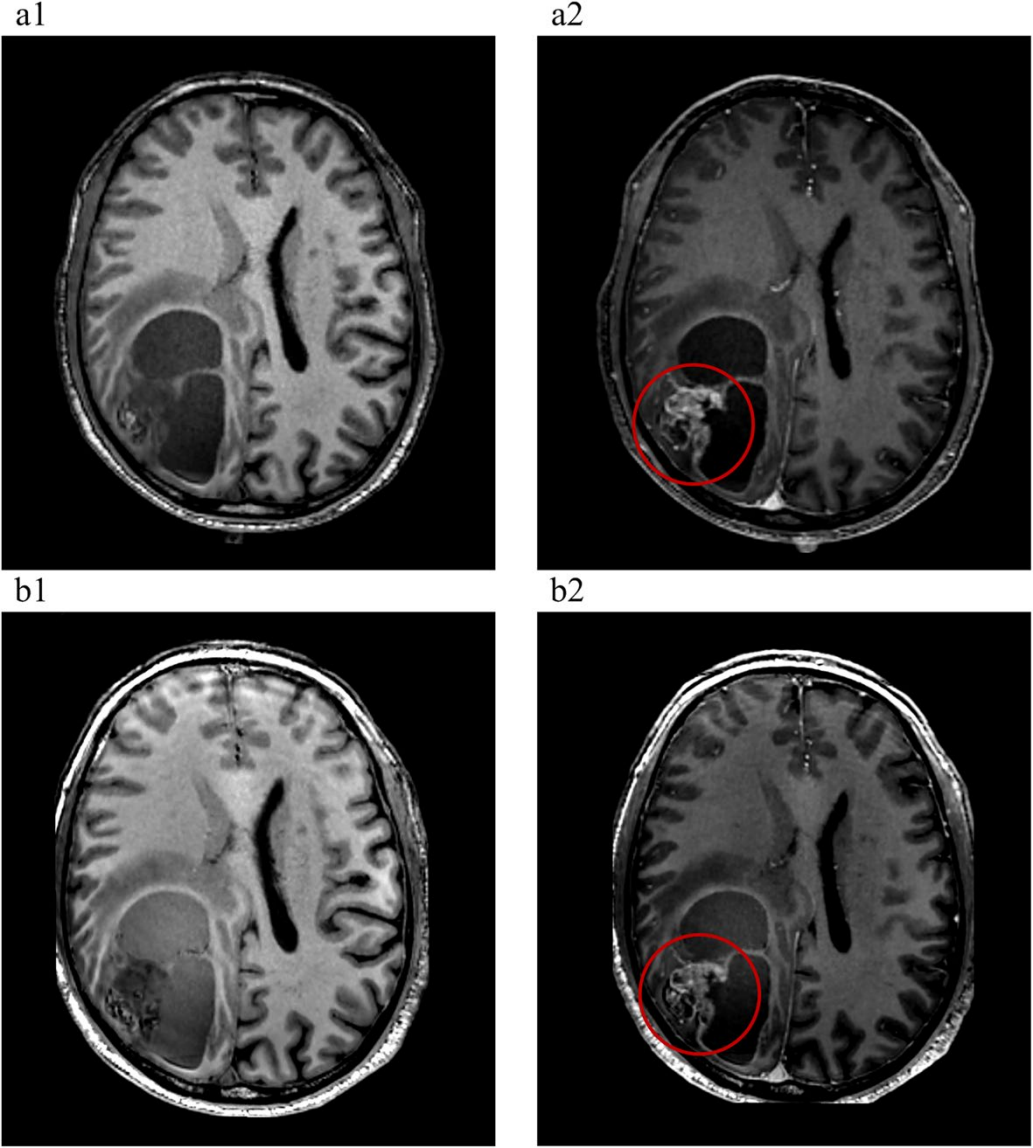
Brain metastases (lung origin), 64-year-old male. (**a1**) T1w MRI without contrast enhancement at 3.0 T and (**b1**) T1w MRI without contrast enhancement at 5.0 T. (**a2**) Full-dose enhanced T1w MRI at 3.0 T and (**b2**) half-dose enhanced T1w MRI at 5.0 T. As depicted within the red circle, the augmentation of the lesion parenchyma exhibits a slightly greater intensity at 3.0 T compared to 5.0 T. However, in terms of delineating the intricacies of the lesion, 5.0 T surpasses 3.0 T due to its employment of a thinner scanning layer thickness and higher resolution

**Fig. 6:**
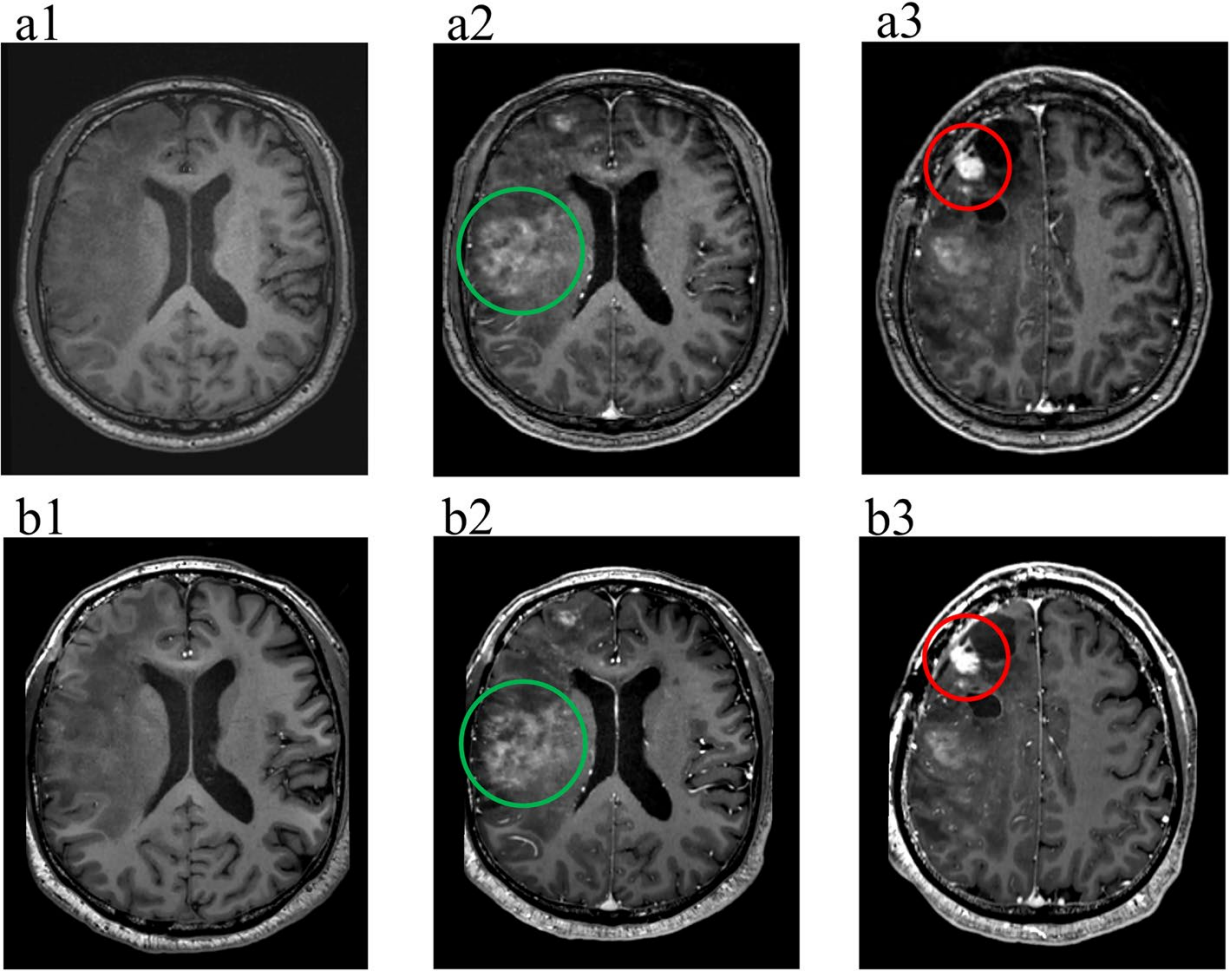
Anaplastic oligodendroglioma, WHO Grade III, 49-year-old male, relapsed after surgery in 2014. (**a1**) T1w MRI without contrast enhancement at 3.0 T and (**b1**) T1w MRI without contrast enhancement at 5.0 T. (**a2**) and (**a3**) Two different slices of full-dose enhanced T1w MRI at 3.0 T. (**b2**) and (**b3**) Two different slices of half-dose enhanced T1w MRI at 5.0 T. For lesions with low-level blood‒brain barrier (BBB) leakage (green circle), the enhancement effect was similar between 3.0 T and 5.0 T, but for lesions with high-level BBB leakage (red circle), the enhancement effect with a half-dose at 5.0 T was significantly better than that with a full-dose at 3.0 T

**Fig. 7:**
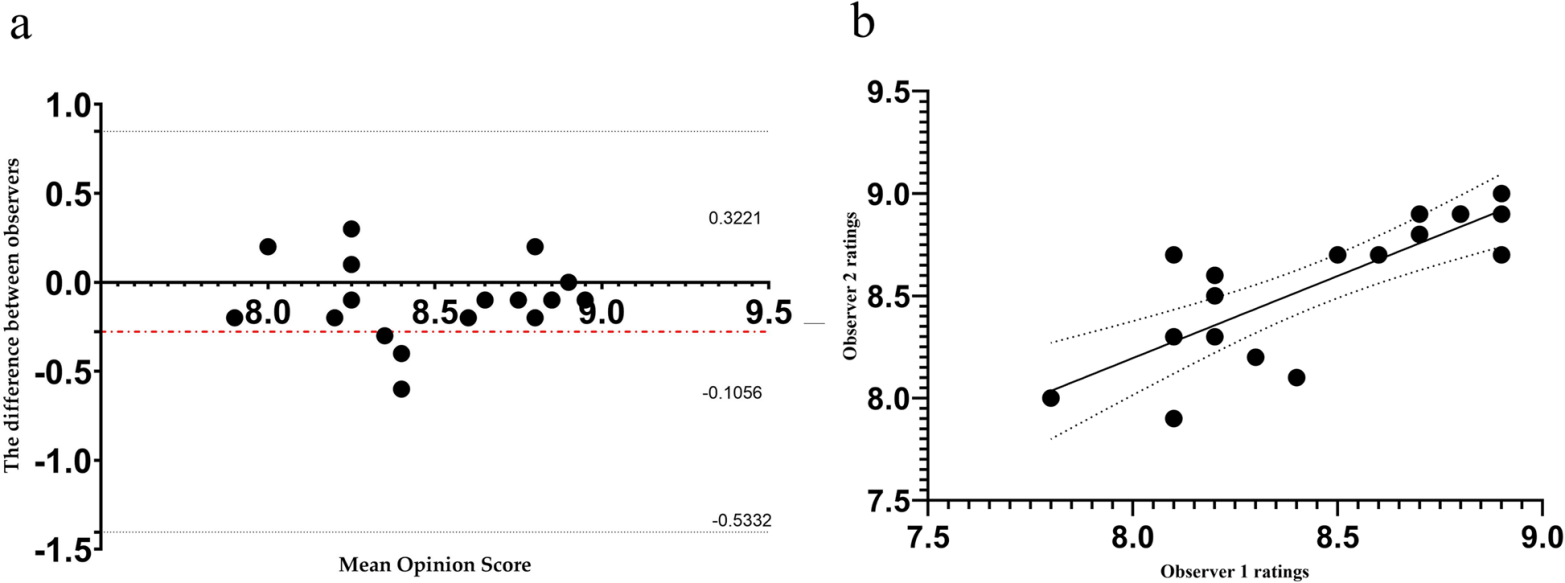
Bland–Altman plot and scatter plot assessing interobserver pairwise agreement for image quality ratings. (**a**) Bland‒Altman plot. The x-axis represents the mean values, and the y-axis represents the difference between the two. The red dashed line shows the mean bias, and the dashed lines show the 95% CI based on the standard deviation of the distribution. (**b**) Scatter plot. The x-axis represents the ratings from observer 1, and the y-axis represents the ratings from observer 2

As shown in Table 4, the average observer ratings were classified as very good or excellent for all the evaluation parameters. Images of different types of tumours acquired at 5.0 T all showed significantly better lesion contours and internal structures than those acquired at 3.0 T, as shown in Figs. 4, 5 and 6. Interobserver agreement was excellent, as assessed by the ICC (ICC >  = 0.89, *p* < 0.01) and Pearson’s correlation coefficient (*r* = 0.80, *p* < 0.01), as shown in Fig. 7.

**Table 4:** Subjective grading of 3.0 T (0.10 mmol/kg) and 5.0 T (0.05 mmol/kg) images

	**Subjective grading**			***P* value**
		**3.0 T**	**5.0 T**	
**Tumour**^**a**^	Delineation	8.1 ± 0.3	8.9 ± 0.3	0.000
	Internal structure	7.8 ± 0.3	8.7 ± 0.4	0.000
	Contrast agent enhancement	8.1 ± 0.3	8.6 ± 0.5	0.037
**Image quality**^**a**^	Grey‒white differentiation	8.1 ± 0.3	8.4 ± 0.6	0.193
	Homogeneity	8.2 ± 0.4	8.2 ± 0.4	0.678
**Artefacts**^**b**^	Motion	8.9 ± 0.3	8.7 ± 0.7	0.343
	Pulsation	8.8 ± 0.4	8.7 ± 0.4	0.343
	Susceptibility	8.5 ± 0.5	8.3 ± 0.4	0.343
Overall sequence quality		8.2 ± 0.4	8.9 ± 0.3	0.001

^a^10-point scale (from 0 = nondiagnostic to 10 = excellent)

^b^10-point scale for artefacts (from 0 = nondiagnostic images to 10 = no artefacts)

## Discussion

The clinical routine for diagnosing brain tumours involves a full-dose contrast-enhanced MRI scan at 3.0 T. In this study, the Gd-based contrast enhancement of brain tumours using a half-dose at 5.0 T and a full-dose at 3.0 T were compared. Both quantitative and subjective evaluation results indicated that 5.0 T MRI with a half-dose of contrast enhancement may be a feasible option to meet the diagnostic requirements in the clinic.

The results indicated that the tumour-to-brain contrast, as reflected by the CNR of lesion/white matter and lesion/grey matter, was significantly greater with a half-dose at 5.0 T than with a full dose at 3.0 T. This finding was consistent with previous studies that compared lesion enhancement between high-field and low-field magnetic resonance imaging (MRI) systems [11]. Moreover, we observed that the CNR increase in some patients and tumour lesions was even greater than the increase in magnetic field strength (i.e., more than 1.7-fold). This might be due to the following two reasons. On the one hand, the effectiveness of the T1-shortening effect of a Gd-based contrast agent increases nonlinearly with the field strength [12]. In vitro experiments indicated that the r1 relativity of Gd-based contrast agents exhibited minimal variations across diverse field strengths [20, 21], and the increased baseline tissue T1 relaxation times at higher fields amplify the relaxation-modifying effect of contrast agents [12]. An increase in tissue T1 values with increasing field strength results in a corresponding increase in relative contrast enhancement. This is due to the combined effects of protein binding, which leads to increased field strength and solvent dependencies, ultimately resulting in notable changes in T1 relaxivity values at higher magnetic field strengths [22]. Prior studies have also shown that the augmentation of channel head coils at elevated field strengths is advantageous for enhancing the signal-to-noise ratio and image resolution of voxels [23, 24]. A 3D-GRE sequence was used in this study, and a similar TR and TE and the same flip angle were set for both 3.0 T and 5.0 T sequences. Therefore, when imaging with similar sequence parameters at both field strengths, grey and white matter may not relax completely and may exhibit a lower-than-expected SI increase at 5.0 T. Since the extensive invasion of glioma may impair the integrity of the blood‒brain barrier (BBB) [25], tumour lesions with more severe BBB disruption would relax almost completely and exhibit high SI at 5.0 T. In addition, a previous study demonstrated that a 3D-GRE sequence was clinically more suitable for detecting brain tumours than other sequences [26]. Hence, the augmented field strength and refined protocols employed in this study resulted in a substantial enhancement in the contrast between tumour tissue and brain tissue at 5.0 T.

In addition to the improved tumour-to-brain contrast, the SNR and CNR of grey matter, white matter, and tumour lesions were significantly greater with half-dose imaging at 5.0 T than with full-dose imaging at 3.0 T. This could be attributed to the greater magnetic field strength (5.0 T vs. 3.0 T) and the greater number of channels of the receiving coils (48 channels at 5.0 T vs. 24 channels at 3.0 T) [27, 28]. Theoretically, a thinner slice or smaller voxel size would result in poorer (lower) SNR and CNR in the same magnetic field due to a decreased amount of aligning protons within the small voxel. The increased SNR and CNR obtained at 5.0 T even with a thinner slice and smaller voxel size indicates the extraordinary benefits of higher magnetic field strength, including clearer images at higher resolutions, which is beneficial for clinical applications [12, 13]. Furthermore, the enhancement of SI at ultrahigh fields, together with the modification of transverse and longitudinal relaxation times, produces enhanced image contrasts that are useful in anatomical MRI applications. We also observed an increase in the grey matter/white matter contrast at 5.0 T versus 3.0 T, which was consistent with previous findings comparing 3.0 T with 1.5 T [29]. A previous study revealed an increased CNR in T1w images at 3.0 T compared with 1.5 T [29]; however, this increase was not found in a previous comparison study of 7.0 T versus 3.0 T [11]. Since B1 field inhomogeneity might influence the CNR [30], this finding at 7.0 T might be because the B1 field inhomogeneity at 7.0 T was more severe than that at 5.0 T and 3.0 T, thus leading to decreased grey matter/white matter contrast [13]. A previous simulation study also revealed that the variation in B1 magnitude was nearly twofold greater at 7.0 T than at 4.0 T [16]. Together with this previous simulation study [31], our findings indicated that 5.0 T might have better B1 field uniformity than 7.0 T and similar uniformity to 3.0 T, thus leading to good grey matter/white matter contrast. Therefore, from a practical perspective, we can conclude that 5.0 T might be superior to 3.0 T in brain tumour imaging, not only because of the greater SNR of brain tissues but also because of better grey matter/white matter contrast than 3.0 T.

One limitation of this study is that only 12 subjects with 16 enhanced lesions were included, and some subjects had only mild BBB leakage. We hypothesized that patients with severe BBB disruption might benefit more from a half-dose at 5.0 T, but the CNR was only slightly improved in lesions with low-level BBB leakage. A previous dynamic contrast-enhanced imaging study suggested that low-level BBB leakage might induce systematic errors in the calculation of measured parameters [32]. In some 3.0 T studies, a double dose is recommended for patients with brain metastases [33]. Thus, our results suggested that the doses of contrast agent might need to be modified for patients with subtle BBB breakdown. In the future, more subjects with different types of brain tumours should be included to increase the generalization of our conclusions. In addition, only one contrast agent (Gadovist) and one type of T1-weighted sequence (GRE 3D) were examined in the current study; thus, our findings might not be applicable to other contrast agents and MRI sequences. However, a previous study suggested that Gadovist is a recommended contrast agent in routine MRI protocols for brain tumours [34], the GRE sequence might be superior to fast spin‒echo sequences [21]. Moreover, we only examined brain tumours, and future studies of tumours in various organs of the body, especially the abdominal area, should be conducted to investigate the full potential of low-dose contrast agent-enhanced MRI on 5.0 T systems. Another limitation of this study is that 3.0 T scanning was performed before 5.0 T scanning for all the subjects due to ethical considerations, and retention of the contrast agent in subsequent scans due to the leakage of contrast agent in tumours would be possible, despite the presence of at least a 24-h gap between the two contrast injection sessions. To rule out this limitation, a precontrast scan was performed to ensure little or no retention of the contrast agent, and the postcontrast images were subtracted from the precontrast images to calculate the SI difference. The precontrast images on the second scan (5.0 T ) showed no significant enhancement. The subtracted SI value was still significantly greater at 5.0 T, as shown in Table 3. Thus, we believe that the cumulative effect of contrast agents on tumour lesions is minimal. In the future, an earlier imaging session of 5.0T may be necessary to support the results of subsequent imaging studies.

## Conclusions

In conclusion, the results of this study suggest that administering a half-dose of intravenous contrast agent in conjunction with 5.0 T MRI could yield improved tumour-to-brain contrast and SNR in enhanced tumour lesions compared to the full dose at 3.0 T MRI. These findings indicate the feasibility of using a reduced dose of contrast agent for the diagnosis of brain tumours in high-field MRI systems.

## Data Availability

The datasets generated and/or analysed during the current study are available from the corresponding author upon reasonable request.

## Abbreviations

MRI: Magnetic resonance imaging
SNR: Signal-to-noise ratio
CNR: Contrast-to-noise ratio
ROI: Region of interest
BBB: Blood‒brain barrier
SAR: Specific absorption rate
SI: Signal intensity
WHO: World Health Organization
TR: Repetition time
TE: Echo time
TI: Inversion time
GRE: Gradient echo
